# Expansive Learning in Practice: A Rapid Evaluation of a Student Nurse Placement Model (Empirical Research Mixed Methods)

**DOI:** 10.1111/jan.70315

**Published:** 2025-10-28

**Authors:** Ailey McLeod, Syka Iqbal, Sophie Moniz, Lucy Mitchinson, Efthalia Massou, Rachel Taylor, Sinead Mehigan, Nikita Hill, Rozalia Dobrogowska, Cecilia Vindrola‐Padros

**Affiliations:** ^1^ Department of Targeted Intervention Rapid Research Evaluation and Appraisal Lab (RREAL), UCL London UK; ^2^ Marie Curie Palliative Care Research Department, Division of Psychiatry, UCL London UK; ^3^ Department of Public Health and Primary Care University of Cambridge Cambridge UK; ^4^ University College London Hospitals NHS Foundation Trust London UK; ^5^ University College London London UK; ^6^ Middlesex University London UK

**Keywords:** evaluation research, nurse education, nursing students

## Abstract

**Aim:**

The aim of this study was to document the process of the implementation and the perceived impact and sustainability of the Expansive Learning in Practice Model and its associated costs to inform future rollout.

**Design:**

A mixed‐method rapid evaluation was conducted, comprising both qualitative and economic workstreams to document the implementation of the Expansive Learning in Practice Model and its associated costs. Semi‐structured interviews (*n* = 44) were carried out with student nurses, student assessors, and staff involved in the delivery of the Model. The qualitative workstream utilised a rapid cycle evaluation approach, where data were collected and analysed in parallel, and preliminary findings were shared with stakeholders as the study was ongoing. The quantitative workstream relied on routinely collected data about non‐staff‐related costs, staff‐related costs, and data on students' participation.

**Results:**

The main themes developed from the qualitative data included the organisation of the Expansive Learning Experiences, the supportive environment, the enhanced learning experience, and capacity building. Participants perceived that the model had a positive impact on student practice (including preparation and confidence) and on student nurse satisfaction. At the end of the programme, it is estimated that the programme will have cost about £523,572.

**Conclusion:**

This model can be used as a framework for hospitals aiming to improve the learning experiences for student nurses. Improvements could be made by increasing staff buy‐in and the streamlining of spoke opportunities. Future studies should focus on evaluating the long‐term impact of the model, particularly the impact on generating student placement capacity. The evaluation also highlights the need for solutions for potential educational staff shortages, which could pose a risk to maintaining sufficient practice placement capacity for student nurses in healthcare settings.

**Impact:**

Study participants perceived an improvement in student nurses' learning experiences and student nurse placement capacity as a result of the implementation of this model.

**Reporting Method:**

The relevant EQUATOR guidelines followed for reporting were the GRAMM guidelines (Good Reporting of a Mixed Methods Study).

**No Patient or Public Contribution:**

The study centred around student nurse and staff experiences.

## 1 Background

Extensive research has been conducted into the best practices for undergraduate nursing education. The importance of incorporating evidence‐based practice into nursing and nursing education has been long recognised (Mackey and Bassendowski 2017), with evidence‐based practice seen as a way to minimise the theory to practice gap within nursing (Cassidy et al. 2021). Previous research emphasised the importance of nurses being critical thinkers in order for them to successfully translate knowledge and evidence into their clinical practice (Falcó‐Pegueroles et al. 2021). Practices such as incorporating reflective practice have been shown to improve both the wellbeing and the competency of student nurses (Contreras et al. 2020). Expansive Learning approaches to nursing education incorporate higher‐level skills including critical thinking and reflexivity along with a focus on evidence‐based practice and learning in real world settings.

### 1.1 Expansive Learning

The Expansive learning framework offers an opportunity to explore learning through work‐based activities (Berragan 2014). The Expansive Learning in Practice Model was informed by Holbery et al. (2019), who introduced a model of coaching which incorporated three phases: Connecting, Establishing and Expanding in Practice Learning. The *Connecting* phase refers to forming an interpersonal connection between student and teacher, ensuring mutual engagement and commitment. The *Establishing* phase involves understanding the students' needs and goals. Together, these two phases create a supportive learning environment and allows students to build confidence. The final phase, *Expanding*, involves the development of higher‐level skills, such as critical thinking, reflexivity and leadership (Holbery et al. 2019). Expansive Learning Experiences are thought to build confidence in students by providing realistic expectations of their programme, developing their professional identity, and developing their skills to provide holistic patient‐centred care (Berragan 2014).

### 1.2 Nursing Curriculum—Moving Towards an Expansive Learning Model (Theory)

The context of nurse education is becoming increasingly complex, with a growing number of students facing academic stress, compounded by separation from support networks, additional learning needs and high expectations of academic achievement (Loureiro et al. 2024; Henderson and Eaton 2013). In 2019, the UK Government set out to recruit 50,000 registered nurses in England by the end of March 2024 (Department of Health and Social Care 2022). Health Education England (HEE) (now ‘NHS England’) invested £15 million to fund additional clinical placements for undergraduate nursing, midwifery and allied health professionals in 2021/2022, with the aim of improving and expanding the healthcare workforce to match future NHS needs (Health Education England 2021). The objectives of this fund were to: (1) provide assurance of ongoing sustainability of placements and required infrastructure for resilience for future years, (2) to support the growth and expansion of practice‐based placements to meet increased student intakes, (3) to maximise practice‐based capacity and efficiency whilst maintaining quality, (4) to ensure that the quality, impact and value of the investment in placement infrastructure, capacity and growth is recognised; and (5) to ensure the development of additional supervision and coaching models to sustain growth (Health Education England 2022).

The nursing curriculum in England currently prioritises an immersion into clinical practice, delivered through Hub and Spoke components of clinical placement. As such, student nurses are allocated a traditional clinical placement (Hub), which is complemented by the possibility of experiencing a different clinical setting under mentor supervision for a limited time (Spoke) (White and King 2015). This model allows student nurses to learn through shadowing of experts in the healthcare environment, with the aim of promoting a holistic understanding of nursing skills and the clinical environment (Morley 2018). However, Berragan (2014) argues that “in the real healthcare setting, learning is a by‐product of care” as the patients' healthcare needs must always take priority over the students' educational needs. Additionally, arguments have been made for a more fluid model of learning which shifts the paradigm from “restrictive” teaching to “expansive” learning as this would foster a more supportive environment for students to learn higher level skills such as problem solving, critical thinking and reflexivity (Fuller and Unwin 2003; Morley 2016; Kaakinen and Arwood 2009).

### 1.3 The Implementation of the Expansive Learning in Practice Model (Practice)

As a recognition of the benefits of the Expansive Learning in Practice Model, the structure of the practice placement component of the nursing curriculum was adjusted to a new model which consists of two interlinking components: the traditional Hub placement and Expansive Learning Experiences. Table 1 gives an overview of the new structure of the practice placement component of the nursing curriculum, under the Expansive Learning and Practice Model. Whilst on Hub placement (which remains as practical experience and shadowing), students access the Expansive Learning Experience Days once a week. The Expansive Learning Experience Days are 7 h of practice‐related activities which count towards their practice hours. During these sessions, Practice Educators (recruited specifically for the Expansive Learning in Practice Model) and Subject Matter Experts (volunteers from the hospitals participating in the pilot) coach students using a range of approaches, such as short presentations, scenario tasks, reflective practice, peer to peer learning and self‐directed learning activities. Through these activities, students expand their knowledge of different specialities and the four nursing pillars: Clinical, Education, Research and Leadership (NHS Scotland 2023). Expansive Learning Experiences also incorporate spoke opportunities. Spoke opportunities are short‐term placements with specialists, for instance, with diabetes, palliative care or respiratory specialist nurses, or with other healthcare professionals, such as educators or pharmacists (White and King 2015).

**TABLE 1:** Structure of practice placement component of the nursing curriculum under the Expansive Learning in practice model.

Component	Description
Hub placement	Traditional clinical placement on a hospital ward.
Expansive Learning Experiences	Additional learning opportunities given to nursing students to provide them with an ‘expansive’ learning experience.
Expansive learning experience days	One day a week where nursing students do not attend their hub placement, and instead learn in a ‘virtual classroom’ environment.
Spoke opportunities	Short‐term clinical placement with specialists.

Expansive Learning Experiences were introduced to enhance the students' learning and to feed back into their clinical learning on the Hub placements and support the achievement of proficiency requirements set out as part of their nursing programme. As students are out of their Hub placement area for their Expansive Learning Experiences, this increases capacity for additional nursing students to be placed in the Hub placements. Facilitating uptake of spoke opportunities, particularly in areas which are currently underutilised in nursing education, may further increase student placement capacity (Health Education England 2022). Additionally, the Expansive Learning Experiences provide an opportunity for students to learn fundamental clinical skills in a safe environment through active learning methods, which prioritises the student's learning needs, as opposed to busy clinical placements (Berragan 2014).

### 1.4 The Clinical Placement Expansion Programme

The Clinical Placement Expansion Programme (CPEP) is an expansion fund provided by HEE to deliver sustainable placement growth in the NHS workforce throughout the academic year 2021/2022 (Health Education England 2022). CPEP funded the pilot programme of the Expansive Learning in Practice Model across five inner city hospitals, with students from five different universities.

We identified four phases of the programme:

Phase 1 (1 July 2021–19 November 2021) involved planning the programme, preparatory work and bid writing.

Phase 2 (20 November 2021–21 February 2022) focused on the recruitment and setting up the programme.

Phase 3 (22 February 2022–6 March 2022) was a two‐week orientation period for the educators.

Phase 4 (07 March 2022–31 October 2022) involved the running of the pilot programme.

## 2 The Study

### 2.1 Aims

The aim of this study was to document the process of the implementation and the perceived impact and sustainability of the Expansive Learning in Practice Model and its associated costs to inform future rollout.

The study was guided by the following questions:

1. What is the programme theory underpinning the model? What are the expected outcomes?
2. What are staff and student nurses' experiences of the programme?
3. What is the cost of running the programme?

## 3 Design and Methods

### 3.1 Design

A mixed‐methods study design was employed to document and understand the implementation and sustainability of the model across five London settings (Palinkas and Cooper 2017). The study was designed using a convergent parallel mixed‐methods approach, where we gathered both qualitative data and quantitative data (data on financial and economic costs) of the implementation from the provider perspective (Mogyorosy and Smith 2005) to answer different research questions. The qualitative workstream involved a focus group and interviews. The economic cost data were provided by the service provider to develop and implement the programme, which included routinely collected data. This study was also designed as a rapid mixed‐methods evaluation, using a formative model to share findings with implementers as the study was ongoing (Skillman et al. 2019). These iterative processes were facilitated by carrying out data collection and analysis in parallel (Clark et al. 2025). A protocol was developed prior to carrying out the evaluation, which guided data collection and analysis.

### 3.2 Theoretical Framework

The Consolidated Framework for Implementation Research (CFIR) was used to inform the collection and analysis of data for the qualitative workstream, particularly in relation to the processes involved in implementation (Kirk et al. 2015). The CFIR domains, specifically those related to the intervention characteristics and the implementation contexts, were employed to define the research questions, shaping interview topic guides and informing categories used during data analysis.

### 3.3 Sampling and Recruitment

We focused on two targeted populations for this study, the staff and the nursing students involved with the Expansive Learning in Practice Model. Data were collected through a focus group of nursing students and through interviews. For the focus group, we used purposive sampling to recruit participants, specifically targeting nursing students at the one hospital where the programme had been implemented the longest. For the interviews, we used purposive and snowball sampling. The inclusion criteria for interviews were staff and students involved in the Expansive Learning in Practice Model at one of the five hospitals. Staff members eligible for inclusion in the interviews were practice assessors, supervisors, or educators working with students in the model. We aimed to recruit nursing students from all the universities, all fields of nurse training (Adult, Children, Mental Health, and Learning Disabilities), and hospitals that took part in the model to capture variation in experiences and views in the interviews. We also aimed to recruit a diverse sample for the interviews in terms of ethnic background and gender to represent the population engaged in the Expansive Learning in Practice Model.

Due to the busy nature of work and education in the NHS, various strategies were employed to recruit participants, which are outlined in Table 2. Students and staff were invited via email, with invitations sent by the Expansive Learning project lead, education teams and university staff. Posters promoting the evaluation were also displayed on hospital wards. Additionally, the education teams raised awareness of the evaluation during the weekly Expansive Learning in Practice sessions. Snowball sampling was also used for recruitment, with participants referring other potential participants to the evaluation team. Those interested in participating in the evaluation contacted one of the researchers (SM or LM) via email. Participant information sheets were shared, and participants signed a consent form prior to the interview. No sampling approaches were used for the economic evaluation of the programme, since we collected all the relevant data available in the five participating hospitals.

**TABLE 2:** Recruitment strategies for each participant group.

Participant group	Recruitment strategies
Staff	– Email invitations – Posters promoting evaluation in wards
Students	– Email invitations – Posters promoting evaluation in wards – Discussions about and presentation of evaluation during Expansive Learning Experience days.

### 3.4 Data Collection

A focus group with nursing students from one hospital was conducted on 30 June 2022. The findings from the focus group were used to identify areas for further exploration in individual interviews. In‐depth, semi‐structured interviews were conducted with student nurses, practice assessors, and supervisors on Microsoft Teams, Zoom, and via telephone from 17 August 2022 to 9 November 2022. These interviews lasted between 11 and 79 min. Three researchers conducted the interviews in parallel, using an interview topic guide. The focus group and all of the interviews were audio recorded and transcribed, and notes were taken by the researchers while conducting the focus group and interviews. The data gathered through transcriptions, focus group notes, and interview notes were entered into data extraction sheets developed by the Rapid Research Evaluation and Appraisal Lab (RREAL sheets), which helped organise and summarise the data in real‐time based on key topics of interest (Vindrola‐Padros et al. 2022). This method allowed researchers to identify gaps whilst data collection was ongoing, maintain consistency between researchers, and rapidly identify key findings of the study to share with implementers (Vindrola‐Padros et al. 2022). An example of a RREAL sheet can be seen in Appendix A.

The interviews explored participants' general understanding of the aims and outcomes of the model, the strengths of the programme, areas for improvement, and the perceived impact on practice. The qualitative evaluation also included observational data, with notes taken during observations also being entered into RREAL sheets. Members of the evaluation team were invited to attend monthly Steering Group meetings and the Practice Educator Hospital Narratives in four hospitals. During these sessions, the Practice Educators shared their experiences with the Expansive Learning in Practice Model in their hospital, including achievements, challenges, and next steps. Additionally, they also observed a Hybrid Workshop attended by various stakeholders from Higher Education Institutions, hospitals across the region, and the student nurse community. The workshop focused on discussing the sustainability and next steps of the model. Emerging findings from the evaluation were shared during steering group meetings and at the Hybrid Workshop. The iterative sharing of emerging findings was beneficial to the implementation of the evaluation as queries and information from the stakeholders helped inform subsequent data collection.

For the economic evaluation of the programme, we used routinely collected data about the non‐staff‐related costs (e.g., costs of printed or and digital material), staff‐related costs (i.e., costs related to the staff involved in the programme), and data on students' participation. These data were provided in collaboration with the programme's data management team. The specific metrics were identified early in the study in consultation with the project leads.

### 3.5 Data Analysis

The research team employed an inductive–deductive approach to data analysis. The analysis was guided by the research questions that shaped the evaluation, while remaining open to new information emerging from the interviews. Using rapid qualitative data analysis methods, the RREAL sheets were reviewed by the research team to identify recurrent topics and themes across participants (Vindrola‐Padros 2021). A list of key findings was generated, and interview notes and recordings were reviewed to create detailed descriptions of these findings. As part of our analysis, we aimed to develop a programme theory based on qualitative data synthesis. This was refined iteratively through interviews, observations, and documentary analysis.

To calculate the staff‐related costs, we used the working hours and the salary band of each staff member involved in the programme, as well as the unit cost information (Curtis and Burns 2015). Non‐staff‐related costs were provided as a total by the data management team of the programme and were not calculated directly by us. To complete the economic evaluation, we calculated the mean cost per student, considering costs incurred and estimated staff‐related costs until the end of the programme (20 February 2023), assuming consistent staff involvement. In addition, we included the contribution of the Steering Group, which had a monthly meeting for an hour from 1 October 2021 until the last date of available data (31 October 2022).

### 3.6 Ethical Considerations

The study was classified as a service evaluation as per the Health Research Authority decision tool (Health Research Authority 2022). Throughout the study, the evaluation team followed ethical principles, ensuring voluntary participation, informed consent, de‐identification of research participants, anonymity, confidentiality of data, and the opportunity to withdraw from the study. All researchers involved were trained in research ethics, information governance, and qualitative research, and the project lead has extensive experience in implementation research, ensuring compliance with ethical standards in the conduct of the evaluation.

### 3.7 Rigour

The study team consisted of both the evaluation team and stakeholders, who were registered nurses involved in the rollout of the model. This collaboration enabled an interdisciplinary and comprehensive evaluation. The evaluation team who conducted the data collection and analysis was all trained in qualitative research and rapid research methods. Regular meetings were held between the two groups to facilitate communication, particularly when queries needed to be addressed to feed back into data collection, and during data analysis. While the evaluation team did not have direct experience working in hospital settings or as nurses, which could have impacted their understanding of some of the themes arising, the collaboration with registered nurses enabled them to obtain a deeper understanding of the context.

## 4 Findings

### 4.1 Participants

The following data were collected as part of Phase 4 of the Clinical Placement Expansion Programme, which involved the running of the pilot programme. Eight students participated in the focus group, and 44 students and staff participated in the interviews, which included 26 students and 18 practice assessors and supervisors. Practice assessors and supervisors included registered nurses with different roles, including Practice Educators and members of the hospital education teams, Clinical Practice Facilitators, and Practice Development Nurses. For more information on participant demographics, see Tables 3 and 4.

**TABLE 3:** Demographics of student nurses.

	Count (*n* = 26)	%
Gender		
Women	21	80.8%
Men	5	19.2%
Age		
Mean (SD)	32.85 (9.89)	
Range	20–51	
Ethnicity^a^		
White British	6	23.1%
White Other	2	7.7%
Black British	1	3.9%
Black African British	2	7.7%
Black African	4	15.4%
Black Caribbean	1	3.9%
Asian British	4	15.4%
Asian	3	11.5%
Hispanic	2	7.7%
Mixed race	1	3.9%
Neurodivergent^b^		
No	19	73.1%
Yes	7	26.9%
Year of study		
1st	3	11.5%
2nd	15	57.7%
3rd	8	30.8%
Field of nursing		
Adult	22	84.6%
Mental health	3	11.5%
Child	1	3.9%
University		
A	5	19.2%
B	5	19.2%
C	1	3.9%
D	1	3.9%
E	14	53.9%
Hospital		
1	3	11.4%
2	4	15.4%
3	11	42.3%
4	5	19.2%
5	3	11.5%

^a^ The participants' ethnicities were grouped by the research team for simplicity.

^b^ Neurodivergent, for example, dyslexia, attention deficit hyperactivity disorder, dyspraxia, autism spectrum disorder, dyscalculia.

**TABLE 4:** Demographics of staff.

	Count (*n* = 18)	%
Gender		
Women	13	72.2%
Men	5	27.8%
Age		
Mean (SD)	44.38 (7.53)	
Range	32–58	
Ethnicity^a^		
White British	1	5.6%
White Other	1	5.6%
Black British	2	11.1%
Black African British	1	5.6%
Black African	5	27.8%
Black Caribbean	2	11.1%
Asian British	1	5.6%
Asian	5	27.8%
Hospital		
1	4	22.2%
2	2	11.1%
3	2	11.1%
4	5	27.8%
5	5	27.8%
Role		
Expansive Learning programme team	2	11.1%
Practice educators	4	22.2%
Education: practice educators, practice education facilitators, education nurse, lead nurse	7	38.9%
Practice: practice assessor, practice development nurse, clinical practice facilitator	5	27.8%

^a^ The participants' ethnicities were grouped by the research team for simplicity.

**FIGURE 1:**
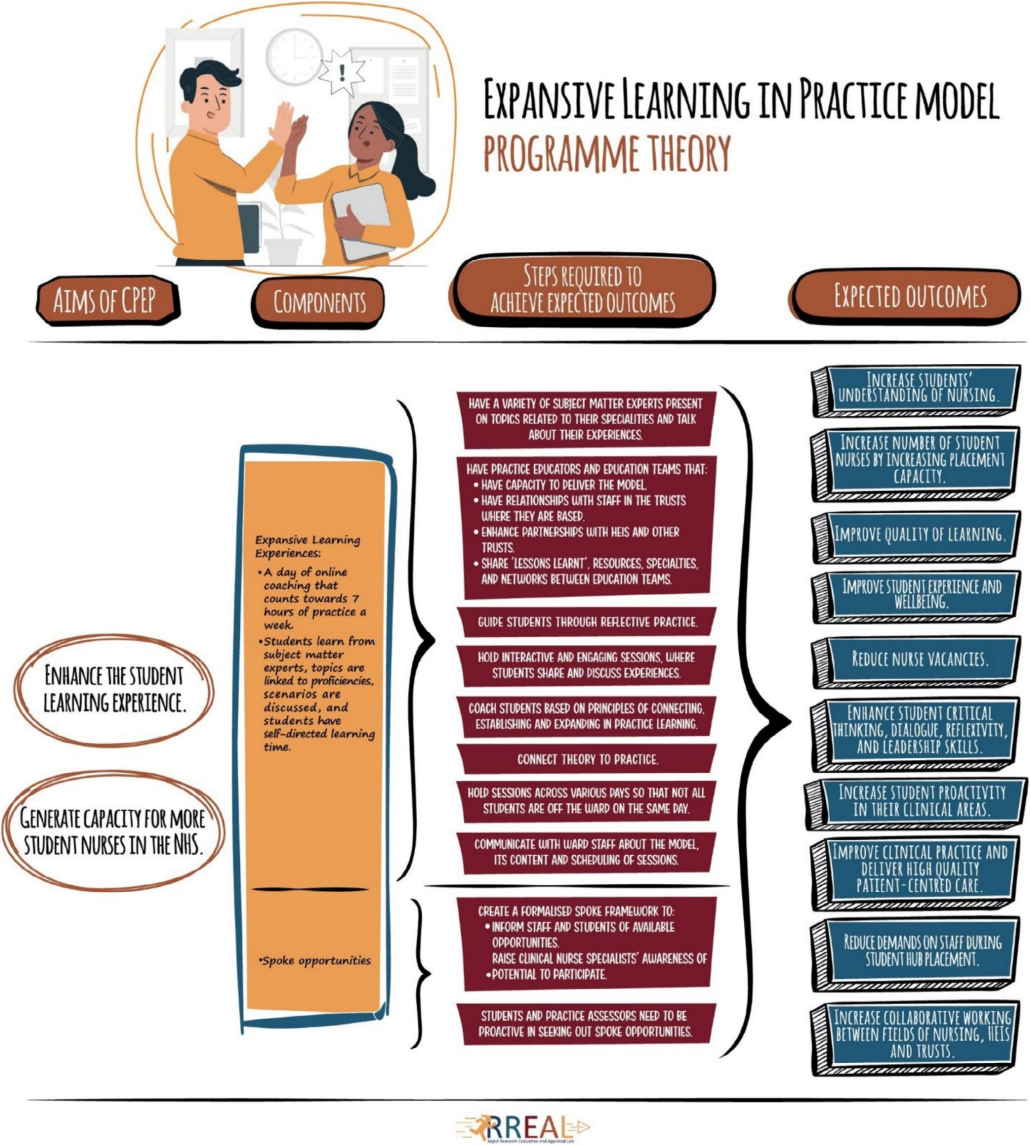
Programme theory.

Four main themes were generated using thematic analysis. Two of these themes related to the facilitators and barriers of the implementation of the model: (Doolen et al. 2016) the organisation of the Expansive Learning Experiences, and (Mackey and Bassendowski 2017) the supportive environment. The other two themes related to the perceived impact of the model on: (Doolen et al. 2016) the students, and (Mackey and Bassendowski 2017) increasing student nurse placement capacity. We also developed a programme theory based on interviews, observations, and documentary analysis (Figure 1).

### 4.2 Programme Theory

As part of our findings, we developed a programme theory that outlines the aims and expected outcomes of the model, along with the key components and steps required to achieve these outcomes (Figure 1). This theory was developed during our analysis of interviews, observations, the focus group, and documentary data. It provided a valuable framework for understanding how the Expansive Learning in Practice Model functioned in real‐world settings and for identifying factors acting as barriers and enablers to its implementation.

### 4.3 Staff and Student Nurses' Experiences of the Programme

The findings from the qualitative workstream have been organised into themes and subthemes, which are outlined in Table 5.

**TABLE 5:** Overview of staff and student nurses' experiences of CPEP.

Themes	Subthemes
Organisation of the Expansive Learning Experiences	Content of Expansive Learning Experiences
	Educational environment of Expansive Learning Experiences
	Structure and application of Expansive Learning Experiences
The supportive environment	A sense of belonging
	A space for reflection
	Practice educators and the education teams
	Staff buy‐in
Enhanced learning experience	Improvement of student practice
	Increase in student nurse satisfaction
	Positive impact on proficiencies
Capacity building	Ability to increase placement capacity of student nurses
	Availability and coordination of resources

#### 4.3.1 Organisation of the Expansive Learning Experiences

The content of the sessions, the educational environment, and the structure and application of the Expansive Learning Experiences emerged as key factors in successfully implementing the model. An engaging and practical approach to organising the Expansive Learning Experiences was crucial for effective implementation.

##### 4.3.1.1 Content of Expansive Learning Experiences

As part of the Expansive Learning Experiences, subject matter experts were invited to share their knowledge. Students felt that this expanded their learning opportunities by allowing them to explore fields of nursing beyond their own pathways of study, offering the chance to learn from specialists, and exposing them to various career options.

Students particularly appreciated being taught topics that related to other fields of nursing, which helped bridge between the different branches of nursing. For example, participants described how learning from mental health specialists provided valuable guidance for students from other fields of nursing on managing patients with mental health issues:

There was a session on mental health and being able to recognise different illnesses, which was really, really useful because I'm an adult nurse and but I meet lots of patients that might have mental health problems. And so it's really good to have a good understanding of the things that we might be meeting. (Student 1)

Students also appreciated the unique learning opportunities the subject matter experts presented, who were often specialists working in busy clinical areas that most students wouldn't typically get the chance to learn from. They valued the opportunity, not only to learn from these experts, but also to hear about their career journeys. This expanded students' perspectives on the various opportunities and pathways available to them for future placements and career directions, as one participant described:

They have specialists that come to talk to us about career pathways and all that. So it gives us a broad idea of the next steps. Like, nursing is not only being on the ward, but other pathways like research and all those things. (Student 2)

Another key finding was that students valued how the topics were directly linked to clinical practice, as the model effectively mapped learning onto the required proficiencies:

It's good to hear from professionals, hear about more general topics you wouldn't necessarily cover, and it links closely to proficiencies. (Student 3)

##### 4.3.1.2 Educational Environment of Expansive Learning Experiences

Participants perceived various factors relating to the educational environment of the Expansive Learning Experiences as beneficial for students. A common theme that arose in the interviews was the importance of the sessions in helping students link theory to practice. The use of scenario‐based tasks helped students reflect on different clinical situations and provided an opportunity for students to think about situations they may not experience in placement. They also enjoyed being able to apply their scenario‐based learning in their placements in clinical areas.

We went through the stages of sepsis identifying how we can manage it […] she was going over it and explaining the steps more in detail than what we go over briefly on the wards. She was going step by step and I was finding out more behind the rationale behind each of these steps. (Student 4)

Furthermore, students were able to request topics, which made them feel that the sessions were relevant to their day‐to‐day practice. Staff participants highlighted that some factors relating to the educational environment of sessions needed to be adjusted during implementation. For example, after recognising that neurodivergent students faced difficulties in traditional lecture formats, the team introduced more structured sessions with clear agendas and visual aids to improve accessibility. Additionally, students with varying learning needs were given more opportunities for one‐on‐one support during group activities. In response to this feedback, sessions were made more interactive, with opportunities for self‐paced learning and tailored activities.

One participant stated:

Increasingly planning activities in a way to engage students with neurodivergent conditions, and taking student feedback to improve [the sessions]. (Staff 1)

Student engagement overall was perceived as varied, with participants highlighting difficulties with facilitating engagement in an online setting.

It can be hard to fully engage the students online. If students have their mic and camera off for the majority of the session, it's not clear how much they are truly engaging. (Staff 2)

The education teams were in the process of adapting sessions in line with student feedback to facilitate engagement, such as by using more breakout rooms, doing quizzes, and encouraging students to keep their cameras on.

They ask them to have cameras on so they can see who is taking part and listening. (Staff 3)

Another staff member mentioned that they were trying to “Make sessions more interactive through simulations of virtual wards.” (Staff 4)

##### 4.3.1.3 Structure and Application of Expansive Learning Experiences

Overall participants felt that the structure of the designated Expansive Learning Experience days and the planning of the sessions were done well, although staff highlighted that Practice Educators would benefit from more resources to assist with the planning of the sessions. One participant stated the difficulties from a Practice Educator perspective included:

Having the capacity to do it all. To deliver the sessions, to deliver the content, and prepare the content. Because its weekly sessions that limits the amount of time that you have to plan ahead, so there's very little time to do that planning and preparation for materials. (Staff 5)

An example timetable can be seen in Appendix B.

Regarding the spoke opportunities, however, it was the lack of structure that acted as a barrier to its implementation. Many students were not aware of spoke opportunities as they were not yet rolled out in their hospitals. This lack of awareness was demonstrated by one participant who, when asked about the spoke placements, responded by asking:

Can I just clarify what's the difference between a hub and a bespoke placement because I wasn't aware that there were two different things? (Student 1)

However, for those who had heard of them, participants highlighted that they would be an extremely valuable experience for students as the spoke opportunities would expose them to different specialities and allow them to gain more specialist knowledge and skills. Nevertheless, they identified barriers to accessing spoke opportunities. Interviewees expressed that it was difficult to obtain spoke opportunities as there was no formalised structure in place to organise them. It relied on student and staff's awareness and proactivity, and some participants were not sure where they could do spoke opportunities or had to rely on having contacts. One participant who had wanted to do a spoke placement but was unable to, explained:

“I couldn't find it within the time I was set, so I couldn't do it.” (Student 4). During the evaluation, the Expansive Learning team was aware these factors would create a barrier to students' participation. A spoke opportunities Framework was being developed which would streamline and compile available services and include a guide on how to use the framework, so that staff and students would understand what was available and appropriate for students.

We're doing our best to arrange spoke placements for student now. So when September comes, when we start a new academic year, then it will be ready… At the moment we're just trying to organize it, you know, setting framework for that spoke placement. (Staff 2)

#### 4.3.2 The Supportive Environment

The supportive environment of Expansive Learning Experience Days was central to facilitating the implementation of the model. Where a supportive environment was not fostered, participants felt that this hindered the rollout. This theme encapsulates the following categories: a sense of belonging; a space for reflection; practice educators; and staff buy‐in.

##### 4.3.2.1 A Sense of Belonging

Expansive Learning Experience Days helped students feel supported as sessions provided regular interaction with staff and other students. It provided them with a space to share experiences, develop relationships, and cultivate a sense of belonging. Students welcomed the opportunities to engage with other students and learn from one another. One participant described feeling reassured by hearing other students share their experiences:

Most of the students share their experience, and I can relate myself to some of this experience. And I feel like I'm not the only one who feels like that. (Student 5)

The regular online group meetings helped students to feel connected to the nursing community, and for international students, assimilate into a new culture. Students appreciated the opportunity to form connections with the education team at their Trust, which one student explained felt like a unique opportunity gained from the Expansive Learning Experiences:

I think another element of it is the connection with the practice education team itself… Having been at another trust and hearing from students at other trusts, I know that they don't necessarily have this support from a practice education team. Like, I think it's unusual that I know their names and who they are, and could identify them. I think a lot of people just wouldn't even have a clue… and I think [the practice education team] have a better understanding of the students and who they are, and their needs and that sort of thing because they're having that regular interaction with them. (Student 6)

##### 4.3.2.2 A Space for Reflection

Expansive Learning Experience Days provided a designated space for students to have reflective discussions. Students were provided with both guidance on how reflection should be done and a designated time to do so. Participants felt that it could be difficult for busy staff in clinical areas to find time to dedicate to teaching, and this made some students reluctant to ask questions on placements. One participant described how the Expansive Learning sessions provide a space to ask questions and understand material in a way that was not always possible on the ward:

When I was in A&E I was asking questions and I was just told, OK, do as you're told. You know, that's just what I was doing. I wasn't relating the concept. I wasn't. I don't understand what I was doing. So I think it was really to me it's really helpful. (Student 7)

Reflective discussions helped students process what they experienced in their clinical areas, with students reflecting on practical lessons learned from their hub placements and as well as talking through more emotionally challenging experiences they'd had on the ward. One student expressed that:

It's an important aspect for us as novice nurses to reflect on what we see in clinical practice, try and make sense of what's going on and share our learning and experience with other students. (Student 8)

##### 4.3.2.3 Practice Educators and the Education Teams

The education teams were highlighted as important to fostering a supportive online learning environment. Students felt that they were encouraging and engaging facilitators. The designated Practice Educators were found to be hugely beneficial for the rollout of the model and were seen as key in curating a supportive space for students to learn and share. Students particularly valued the effort they made to engage and connect with them and felt that they were approachable and encouraging. One participant described the impact practice educators and the education team had on their experience:

The whole reason why you feel like engaging because you feel included… the rest of the team as well the very kind and supportive and never been aggressive at one point or never been rude, always always are willing to answer any questions, always sign posting us to whatever we need to go and get answers from, you know so that, that that makes a big impact. (Student 9)

They helped create a non‐judgemental space where students could share their views and feel comfortable asking questions and sharing reflections. From the point of view of staff participants, the Practice Educators were considered helpful in the organisation, coordination, and general management of the model.

##### 4.3.2.4 Staff Buy‐In

Lack of staff buy‐in hindered the implementation of the model. Some felt that lack of buy‐in impeded students' learning in clinical areas as staff missed opportunities to implement their learning. There were various factors that led to lack of buy‐in. Some nurses were seen as traditionalists who valued practice experiences in the clinical areas over other forms of practice learning. Many participants found that ward staff were not aware of the model, with one participant describing the difference in staff awareness at their latest placement compared to their first ward placement:

I think actually [my latest placement has] probably been the most successful because they're very aware of this Expansive Learning program, that we have this day on a Thursday we're at online…They've got a better understanding of how the model works. (Student 8)

Some students did not see this as a problem as they felt that once informed, staff accepted it and no obstacles were created. However, other interviewees highlighted that better communication was needed between the hospitals, Higher Education Institutions and the Expansive Learning teams. Incorrect terminology was also perceived to contribute to lack of staff buy‐in. The placement model was seen by some as a virtual placement or home study day, which participants felt influenced the perception of the model as separate from students' clinical placement (instead of understanding that it formed part of a cohesive placement experience). Participants believed that increasing staff's awareness and adjusting the language used, such as describing it as ‘coaching’ rather than a ‘study day’, would reinforce the link to clinical practice and increase acceptance.

#### 4.3.3 Enhanced Learning Experience

When reflecting on the impact of the model, the main categories that arose from the interviews were: improvement of student practice, increase in student nurse satisfaction, and a positive impact on proficiencies.

##### 4.3.3.1 Improvement of Student Practice

The main perceived impact was that the model improved students' practice, with participants highlighting a variety of contributing factors. Interviewees felt that students were better prepared for practice because of the model, with students explaining that the Expansive Learning Experiences acted as a bridge between university and placement. They better understood what was expected of them, and it provided a designated time to reflect on practice. One participant described how an Expansive Learning Experience day helped them to understand more about the sickle cell patients they had been seeing frequently during their ward placements:

I've always been asking the staff there about the sickle cell patients we tend to have in A&E, patients who come in every two days…I kept on asking myself. But why are they here? You know, you're treating them. [Expansive Learning] gave me more of a broader image and now I understand… why they're constantly in A&E. (Student 10)

Improvement of students' practice was also attributed to improvement of students' confidence, with one participant stating that:

[Expansive Learning] really made me more confident because I know the theory behind what I'm doing on the ward. (Student 2)

Discussing topics and experiences before going into practice made interviewees feel that students were better equipped for placement, which meant they could provide better care in their clinical areas. Participants believed the model increased students' proactivity in their clinical areas, as the model encouraged them to be involved, and by feeling more equipped for practice meant they did not have to wait to be told what to do. However, there were a few participants who felt that students missed out on clinical experiences and learning opportunities whilst at the Expansive Learning Experiences. In particular, a few staff members were concerned that the online format was not beneficial to all students and felt that being physically in their clinical areas was necessary for them to learn. One staff member relayed how students expressed a desire for more time in the wards instead of spending time in Expansive Learning:

Some students thought they need more clinical time. instead of hopping on a whole day of [Expansive Learning], they would prefer to be in a ward. (Staff 4)

Participants perceived that the model improved students' critical thinking and communication skills. They felt that being provided with a designated space to reflect on their experiences led to deepened clinical reflections. Students particularly appreciated discussing challenging experiences they had faced in their clinical area and learning from them. For example, discussing and reflecting on professional communication improved their communication skills in their clinical areas and gave some students the confidence to speak up when they faced an issue with staff members.

The ward manager was like assigning my other colleague to do 1 to 1 with one of the patients… so I was like wait hold on a minute… ‘We're here to train. We're here to learn. We're not qualified to do 1 to ones.’ And because we had discussed this previously [during an Expansive Learning Experience], it gave me the confidence to speak up. If I hadn't, she would have done 1 to 1, the whole shift. (Student 9)

However, whilst some students felt that being better equipped for practice led to an improved relationship with staff in clinical areas, others felt that it created tension between them. Participants either believed this was due to staff resistance to a new way of learning, or due to difficulties faced by clinical staff when student nurses are attending Expansive Learning sessions instead of being on the ward.

A negative is there's an allocated day [when all student nurses attend Expansive Learning Experiences]. The staff they really rely a lot on students because it does help with the students being there and assisting. (Student 4)

##### 4.3.3.2 Increase in Student Nurse Satisfaction

Participants believed that the practice model improved student nurse satisfaction. One recurring recommendation from both students and staff participants was to expand the model to include all nursing students, reflecting how valuable they perceived the model to be on the students' nursing experience. This was demonstrated in a comment from a staff participant:

“Because all of the students were not involved [in expansive learning] there was that feeling of by students who were not involved of being deprived.” (Staff 6) Some students indicated that the dedicated space to reflect and learn from nurses, combined with the increased preparation for practice, improved their wellbeing. Others highlighted that it was due to having their voices heard and feeling cared for. Some interviewees believed that increased student satisfaction would lead to increased nurse satisfaction and retention in the long run.

A lot of students enjoy sessions no matter what they're about. Even if it's just one or two hours just to consolidate or go over everything… just saying whatever has happened throughout the day, If it's good or bad. So I think it's a nice atmosphere to just reflect confidentially. (Student 4)

##### 4.3.3.3 Positive Impact on Proficiencies

Most interviewees felt the Expansive Learning in Practice Model had a positive impact on students' proficiencies, with one participant stating that because of Expansive Learning sessions:

“I'm now confident with my proficiencies. I don't know what I would have done [without the Expansive Learning] because in my last placement with the proficiencies, I always had to try and go back and check maybe with Google.” (Student 11) Some participants felt the sessions improved their skills overall and created a more consistent level of skill among the students. Participants appreciated that discussing the proficiencies based on what was learnt during the Expansive Learning Experiences could be used as evidence for proficiencies. Students felt the sessions helped them apply skills confidently in their clinical area.

If we don't do some of the proficiencies in the [hub] placement and we cover them in our Expansive Learning sessions then we can tell our assessors, “we discussed this, you can ask me questions and then you can sign them off.” (Student 13)

However, some participants felt that the sessions could focus more on clinical skills.

So I think [during Expansive Learning Experience Days] they should mainly focus on our helping us with the clinical skills like not just communication team management but in terms of medication, drug calculation, different types of medication. (Student 12)

Whilst some students felt that practice assessors were very helpful in checking off skills, some interviewees found it difficult to demonstrate the proficiencies they learnt during the Expansive Learning Experiences due to a lack of availability of practice assessors and supervisors in their clinical area.

I had practice assessor, but she's on annual leave so I wouldn't see her until I finished placement. But then [I was told] ‘don't worry, we will assign you to others and you can talk to them, communicate with them’, but they are mostly charge nurses, so I won't get to spend some days with them [as they are very busy]. (Student 4)

#### 4.3.4 Capacity Building

The main theme emerging from the interviews regarding the potential of the Expansive Learning Model to increase placement capacity for student nurses centered on both the opportunities and challenges associated with its implementation. While some participants felt that the model had the potential to increase capacity, others identified significant barriers that could hinder its sustainability. These barriers were mainly related to resource availability, coordination challenges, and the reliance on designated staff roles. Below are the subthemes that illustrate these discussions.

##### 4.3.4.1 Ability to Increase Placement Capacity of Student Nurses

Some participants believed that the model was generating more placement capacity. Staff at one hospital believed that capacity was increasing, as they were able to provide Hub placements that had not taken on students before the model. Staff based at another hospital, where the model had been rolled out the longest, felt that it was succeeding in generating capacity, with one participant stating that:

[Expansive Learning] does have great potential to increase capacity really easily without making people feel too burdened. (Staff 1)

One interviewee at this hospital believed that there were more students on the wards and less congestion as the model made the number of students manageable, which could reflect the benefit of time in embedding this as the cultural norm at this hospital.

##### 4.3.4.2 Availability and Coordination of Resources

Many staff felt that students attending the Expansive Learning Experiences on the same day did not generate capacity to have more students on the ward. Having all the students out on 1 day did not provide enough time to take on new students. Some interviewees believed that it created more pressure on staff on the ward who had to deal with the students' absence and then subsequently deal with all the students on the ward at the same time. One participant expressed the difficulties that can be caused by having all the students attend the Expansive Learning Experience on the same day every week:

Having the students attend these online sessions can be really hard because if the practice assessors is working on that that Wednesday and the student is not here that day, it does have an impact. (Staff 7)

To address this, participants indicated that sessions should be held across various days. However, many voiced concerns over being short staffed. Some were uncertain if the educations teams would have the capacity to deliver more sessions during the week. There were concerns that they would need more practice assessors on the ward to meet the demand of an increase in students.

If we had first year, second year third year [student nurses] all [attending Expansive Learning Experience] on the same day, it is not going to increase capacity. It has to be spread out throughout the throughout the week…And then I would just worry whether we have the capacity to do that in, in our team. (Staff 8)

Other participants believed that this was not an issue, as dividing students up and spreading their attendance to the Expansive Learning Experiences across various days would reduce the impact on practice assessors. If rotas were done in a way that students were spread out during the week at Hub placements, this would free up more space and would make it manageable for practice assessors on the ward.

[Student nurses] should work at 24 h pattern seven days a week including weekends and nights, days, long days and so on. And then it would work because it would mean that you're able to spread out the numbers across the seven day week and actually have very little students on the board, which is quite nice for everyone and to enhance opportunity and space for learning for deeper learning. (Staff 1)

Another concern regarding capacity was that the sustainability of the model would be compromised without having the designated Practice Educators in future roll outs of the model, as they were funded as part of the pilot programmes. Staff who were part of the education teams felt this would increase their responsibility and were concerned about not having the capacity to take on this additional workload. For the model to be sustainably rolled out without Practice Educators, participants believed that it would be necessary to continue working together across the system to create and share resources to support the education teams.

[To role out the model without Practice Educators] I would need some good liaison with the current Practice Educators to use some of their resources because they have existing resources already. And I suppose that would need to really be shared with me, so I don't have to start everything from scratch. But it is quite daunting because you know it's going to take you off of your current role for probably two days a week. (Staff 9)

### 4.4 The Costs of the Programme

The details about the human resources needed in each phase of the programme, along with their estimated costs, are presented in Table 6. We gathered financial and economic costs of the implementation from the provider perspective (Mogyorosy and Smith 2005). The table shows calculations about the cost of planning and setting up the programme, as well as calculations about the cost of running the programme. A prediction of the staff‐related costs until the end of the programme is also provided in the same table. According to our prediction, until the end of the programme, the staff involvement costed an additional £151,585 while the final contribution of the Steering Group added £1245 to the costs. At the end of the programme, it is estimated that the programme cost about £523,572, including the staff‐related and the Steering Group‐related costs.

**TABLE 6:** Staff‐related costs of the programme.

	Number of staff members involved	Salary band of the staff	Working hours per week	Total staff‐related costs^a^	Prediction until the end of the programme
Phase 1: planning phase	1 Host/Sponsor and Head of Nurse Education	Band 8d	1	£4741.5	NA
	1 Programme Director	Band 9	0.5		
	1 Assistant Chief Nurse	Band 8b	0.5		
	1 Senior Nurse Undergraduate Education	Band 6	0.5		
Phase 2: setting up and recruitment	1 Senior Nurse Undergraduate Education	Band 6	15	£11,520.8	NA
	1 Host/Sponsor and Head of Nurse Education	Band 8d	1		
	1 Project manager officer	Band 6	0.75		
Phase 3: 2‐weeks orientation for the educators	5 x Educators	Band 6	37.5	£20,110.5	NA
	1 Project manager officer	Band 6	0.75		
	1 Senior Nurse Undergraduate Education	Band 6	15		
	1 Host/Sponsor and Head of Nurse Education	Band 8d	1		
Phase 4: running the pilot programme	1 Host/Sponsor and Head of Nurse Education	Band 8d	1	£329,927.5	£151,585
	1 Senior Nurse Undergraduate Education	Band 6	15		
	1 Project manager officer	Band 6	0.75		
	4 x Educators	Band 6	37.5		
	1 Nurse	Band 6	30		
Steering group	1 Lecturer in nursing	Band 7^b^	0.25	£4442.2	£1245.2
	1 Ward manager	Band 7	0.25		
	2 Head of Education	Band 8d	0.25		
	1 Associate Professor/Director of the programmes	Band 8^b^	0.25		
TOTAL	—	—	—	£370,741.5	£523,571.6

^a^ The total staff‐related costs have been calculated taking into consideration the duration (in weeks) of each phase.

^b^ These are academic scales, not NHS.

The non‐staff‐related costs included hidden costs of the project and transparent costs, which were costs related to the setting up and developing of resources for the website. This was not funded through the bid. These costs were approximately £11,500 and it is estimated that there will be an additional annual cost for updates that will be equal to £1200. No paper documents have been produced; all sessions, advertising and promotion have been done digitally. No non‐staff‐related costs during the programme implementation have been reported so far; however, the above‐mentioned amount of £1200 should be included when the future implementation of the programme is considered.

Up to the date that this analysis was conducted (i.e., January 2023), 1315 students from all involved hospitals had participated in the Expansive Learning Placement Model. This was an average running cost of £254 per student, or £270 if we include the cost of the 2‐week orientation of the educators. This cost has been calculated considering the staff‐related costs and the contribution of the Steering Group during phase 4. An oversimplified estimation about the programme's efficiency is that about 15% of the students participating in the programme take jobs at their sites.

## 5 Discussion

This evaluation found that the implementation of the Expansive Learning Experience Days generated a supportive environment, which was essential for the model's success. Participants highlighted that the model prepared students for practice by bridging the gap between university learning and clinical placements. This preparation included a clearer understanding of expectations and designated time for reflection on practice. This may reflect the growing importance of coaching‐based models of teaching, which represent the work, skills, and support that student nurses need.

The evaluation highlighted that the connection with practice supervisors is important, as we found that the lack of staff buy‐in in clinical areas hindered the implementation of the model. Some participants believed that the lack of staff support led to missed opportunities for learning in clinical areas and created tense relationships with staff (due to staff resistance to adapting to a new way of learning). Staff support also emerged as an important theme in an evaluation done on the Collaborative Learning in Practice (CLIP) model (Hill et al. 2020). During CLIP, students were guided by a coach and worked with other students during their placement as a method to enhance their learning. The evaluation found that “a culture which was receptive to change” was important to the successful implementation of the model (Hill et al. 2020, 3).

For the Expansive Learning in Practice Model, our findings suggest that connection with ward staff (including practice supervisors) is important to create a space where learning takes place. However, this connection was not as central as the connection with the education teams and other students. Our findings showed that the approachable, non‐judgmental, and encouraging Practice Educators were important for the successful implementation of the model, as it created a space where students could ask questions and discuss openly. Students perceived these sessions to cultivate a sense of belonging, as these provided a time to share and learn from other students. The importance of a sense of belonging for clinical learning is supported by a study conducted by Levett‐Jones and Lathlean (2008).

Our findings also align with a virtual placement model rolled out to health and social care students as a way of responding to the circumstances of the COVID‐19 pandemic in the UK (Taylor and Salmon 2021). This study found that the online design of the Peer‐Enhanced E‐Placement (PEEP) not only provided a practical response to the COVID‐19 pandemic, but also acted as a way to increase peer engagement and learning and collaboration opportunities (Taylor and Salmon 2021). Similarly to PEEP, the Expansive Learning Experiences took place virtually, highlighting the positive benefits virtual placements can have on students' learning. Therefore, the results from our study suggest that establishing connections with a wide network of staff and students is important for fostering clinical learning. Although a lack of connection with a practice supervisor may affect learning, it may not be as central if other relationships create a sense of belonging and a supportive space for learning.

The evaluation showed that the coaching model considered students' learning needs, including those of neurodivergent students. Adaptation of the sessions to meet the students' learning needs was ongoing as the model was being rolled out. Importantly, the findings also showed that the Expansive Learning Experiences created a space outside the busyness of practice where students could ask questions, something which students felt they often did not feel comfortable doing in their clinical areas. This was suggested to have a positive influence on student nurse satisfaction, as some students attributed their increased satisfaction to having their voices heard and feeling better equipped for practice. A study conducted by Collard et al. (2020) showed that student nurse satisfaction influenced nurse retention. This suggests that in the long term, the implementation of the first two stages of the Expansive Learning in Practice Model may contribute to higher nurse retention rates.

Study participants perceived that the model better prepared students for practice by improving their confidence, critical thinking, and communication skills. Participants felt that the sessions deepened their clinical reflections as they had space to reflect on their clinical experiences and process their experiences both practically and emotionally. The importance of having a dedicated space to reflect is supported by the findings of a simulation model that highlighted that a benefit of the model was that it provided a way for students' needs to be placed at the centre (Berragan 2014). In health care settings, the patients' care needs are placed over the students' educational needs (Berragan 2014), with the clinical environment diminishing the possibility for students' personal learning and reflection due to the priority of patient care.

Pearce et al. (2022) highlighted that a challenge for many hospitals in the UK is to find ways to increase student numbers without negatively impacting the quality of their learning experience. The evaluation showed that some participants perceived more capacity on the wards, but many participants felt that the Expansive Learning Experiences needed to be rolled out across a variety of days in order to create capacity successfully. Whereas the results of the CLIP model showed that staff shortages in the clinical areas had a negative impact on student learning (Hill et al. 2020), study participants believed that the Expansive Learning in Practice Model could provide a way for students to receive quality education without being negatively impacted by staff shortages. Although a potential lack of capacity within the education teams may be a challenge, the model could provide a solution to shortages of staff in clinical areas. This could be mainly achieved through the implementation of Expansive Learning Experience Days as an additional educational opportunity for student nurses, as well as the ability for these online sessions to reduce the number of student nurses in clinical areas at one time. The evaluation also indicated that approximately 15% of the students taking part in the programme would take employment roles at their site, providing some preliminary evidence about the sustainability of the model. It is recommended that the model distributes the Expansive Learning Experiences over various days of the week, and that subsequently capacity is evaluated.

## 6 Limitations

One significant limitation was the difficulty in recruiting participants, as both staff and students had demanding schedules amidst ongoing staff shortages and industrial action. This resulted in a smaller sample size that was less diverse than originally anticipated. There is a risk that some staff and student experiences were not captured, and the findings may reflect the perspectives of only a subset of those involved. Additionally, while we could not compare across all the separate hospitals, the common themes identified suggested that the findings will be relevant to the future roll‐out of the model in all hospitals.

Another limitation arose in relation to the focus group. While we initially planned to involve students from all hospitals implementing the model, as well as practice assessors and supervisors in the focus group, difficulties with recruitment meant this was not possible. Recruitment difficulties for the focus group were most likely due to industrial action at the time, disrupting work hours, as well as understaffing and busy schedules. The study was carried out in a small sample of sites, limiting the generalizability of the findings.

In addition, given the relatively recent rollout of the model and the rapid nature of the evaluation, we were unable to assess the long‐term impact of the model. Longer‐term impact of the model, including on nurse retention rates and student capacity, should be evaluated in the future. Finally, in our quantitative workstream, we made a series of assumptions regarding the number of students participating in the programme to explore its efficiency. Assumptions about students remaining at their sites may influence conclusions regarding the model's cost‐effectiveness.

## 7 Conclusion

Our evaluation suggests that study participants perceived an improvement in their student learning experience as a result of the Expansive Learning in Practice Model. The factors identified as facilitators in implementation were: an engaging and practical organisation and a supportive environment. The factors acting as barriers were: staff buy‐in and the organisation of spoke opportunities. Potential adopters of the programme would need to assess the costs of running the programme in relation to the potential percentage of students taking employment roles at their site as a result of the programme. Future research should focus on generating student placement capacity.

## Author Contributions

Made substantial contributions to conception and design, or acquisition of data, or analysis and interpretation of data: A.M., S.M., L.M., E.M., R.T., Si.M., N.H., C.V.‐P. Involved in drafting the manuscript or revising it critically for important intellectual content; given final approval of the version to be published. Each author should have participated sufficiently in the work to take public responsibility for appropriate portions of the content; agreed to be accountable for all aspects of the work in ensuring that questions related to the accuracy or integrity of any part of the work are appropriately investigated and resolved: A.M., S.I., S.M., L.M., E.M., R.T., Si.M., N.H., R.D., C.V.‐P.

## Ethics Statement

The study was not eligible for Health Research Authority (HRA) approval, as it was classified as a service evaluation as per the HRA decision tool.

## Conflicts of Interest

Si.M. and N.H. were involved in the design and implementation of the Expansive Learning in practice model. The other authors declare no conflicts of interest.

## Data Availability

The data that support the findings of this study are available from the corresponding author upon reasonable request.

## Appendix A Example RREAL Sheet

**Table:**

Category	Findings
Perception of purpose of the project	
Perception of intended benefit to students	
Perception of intended benefit to staff	
Experience of hub placement	
Hub placement—benefits	
Hub placement—challenges	
Experience of spoke opportunities	
Spoke opportunities—benefits/facilitator	
Spoke opportunities—challenges	
Overall experience of Expansive Learning Experiences	
Expansive Learning Experiences—benefits	
Expansive Learning Experiences—challenges	
Positive impact of Expansive Learning Experiences on students	
Negative impact of Expansive Learning Experiences on students	
Impact of model on getting hours checked off	
Impact of model on getting proficiencies checked off	
Positive impacts on staff	
Negative impacts on staff	
Perception of staff relationship to model (awareness? supportive? issues?)	
Parts of model that should stay the same	
Suggested improvements	
Additional comments	

## Appendix B Expansive Learning Timetable: Mental Health Day

### Fields of Nursing: Mental Health, Adult and Children Nursing

**Table:**

09:30–09:45	09:45–10:00	10:00–11:00	11:00–11:15	11:15–12:15	12:15–13:00	13:00–14:00	14:00–14:15	14:15–14:45	14:45–15:15	15:15–16:15	16:15–16:30
Registration, etiquette and updates	Introduction to Expansive Learning mental health day and ice breaker	The importance of understanding mental health in all fields of nursing (holistic patient care)	Break	“Compassion in the eyes of a service user”	Lunch	Is dementia a mental health illness? *care for patients with dementia* vs. *delirium*	Break	Bite‐size masterclas‐ses: depression and self‐harm	Bite‐size masterclas‐ses: psychosis	Mental health resiliency for students	Evaluation and Close
	Practice educators										

### Mental Health Nursing, Adult and Child (Part 1, 2 and 3)

PLPAD Assessment of Proficiencies—Appendixes A and B (The learning you gain in these Expansive Learning sessions will support the achievement of the below proficiencies in practice)

**Table:**

Part 1	Assessment of performance in episode of care	Participates in assessing needs and planning person‐centred care 1, 3	Participates in providing and evaluating person‐centred care 4, 5, 6, 7, 8	Participates in improving safety and quality of person‐centred care 24, 26, 28, 29
Part 2	Assessment of performance in episode of care	Participates in assessing needs and planning person‐centred care with increased confidence 1, 2, 3	Participates in delivering and evaluating person‐centred care with increased confidence 5, 6, 7, 8	Participates in the procedures for the planning, provision and management of person‐centred care with increased confidence 22, 29, 30, 32, 33
Part 3	Assessment of performance in episode of care	Confidently assesses needs and plans person‐centred care 2, 4, 5, 6, 7, 8, 9	Confidently leads and mana‐ ges person‐centred care and working in teams 18	Confidently coordinates person‐centred care 25, 26, 29
